# Supplementary material for: RAC1B: A Guardian of the Epithelial Phenotype and Protector Against Epithelial-Mesenchymal Transition
Source: Cells. 2019 Dec 4;8(12):1569. doi: 10.3390/cells8121569 (PMC6952788; doi:10.3390/cells8121569)
Supplement: Supplementary file 1 [file cells-08-01569-s001.pdf]

Supplementary Figure S1

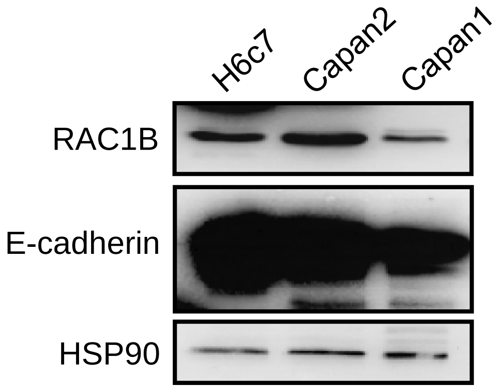

Figure S1. Immunoblot analysis of RAC1B and E-cadherin in H6c7 and Capan1 cells. Protein lysates from the benign human pancreatic ductal epithelial cell line H6c7, the human PDAC-derived line Capan1, and Capan2 as control (see Figure 1A), were fractionated by SDS-PAGE, blotted and incubated with antibodies to RAC1B, E-cadherin, and HSP90 to verify equal loading.

Supplementary Figure S2

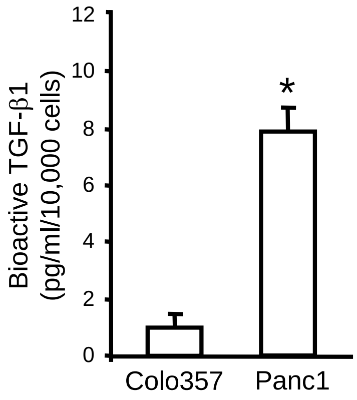

Figure S2. Concentration of bioactive TGF-β1 in culture supernatants of Colo357 and Panc1 cells as measured by ELISA. Data represent the mean ± SD of three experiments. The asterisks indicates a significant difference.

Supplementary Figure S3

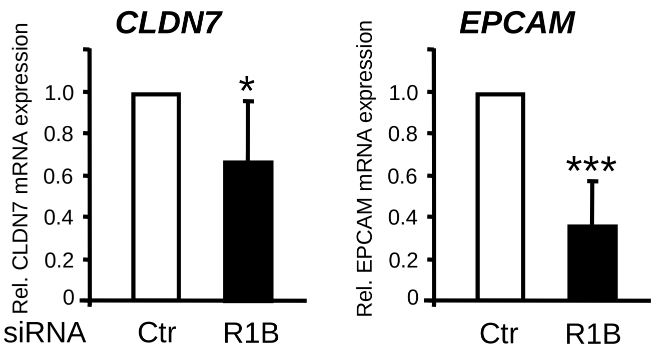

Figure S3. Effect of RAC1B knockdown on *CLDN7* and *EPCAM* expression in Panc1 cells. Panc1 cells were transiently transfected twice with 50 nM each of control (Ctr) siRNA or RAC1B siRNA (R1B). Fortyeight h after the second transfection cells were processed for RNA isolation and qRT-PCR analysis. Data represent the TBP-normalized mean ± SD of three experiments. The asterisks indicate significance.

Supplementary Figure S4

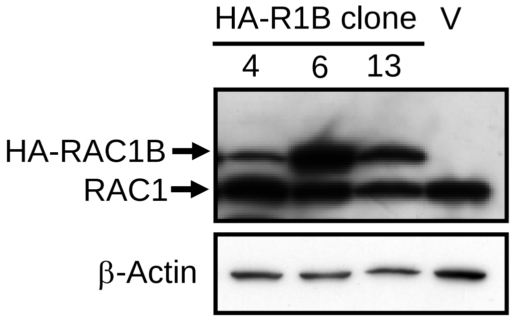

Figure S4. Immunoblot detection of RAC1B and RAC1 in Panc1 cells stably expressing a HA-tagged version of RAC1B. Crude protein lysates of three individual clones of Panc1-HA-RAC1B cells as well as of empty vector (V)-transfected control cells were fractionated by SDS-PAGE, blotted and incubated with an anti-Rac1 antibody that recognizes both RAC1B (upper band) and RAC1 (lower band). Incubation of the same blot with an antibody to β-actin served to verify equal loading.

Supplementary Figure S5

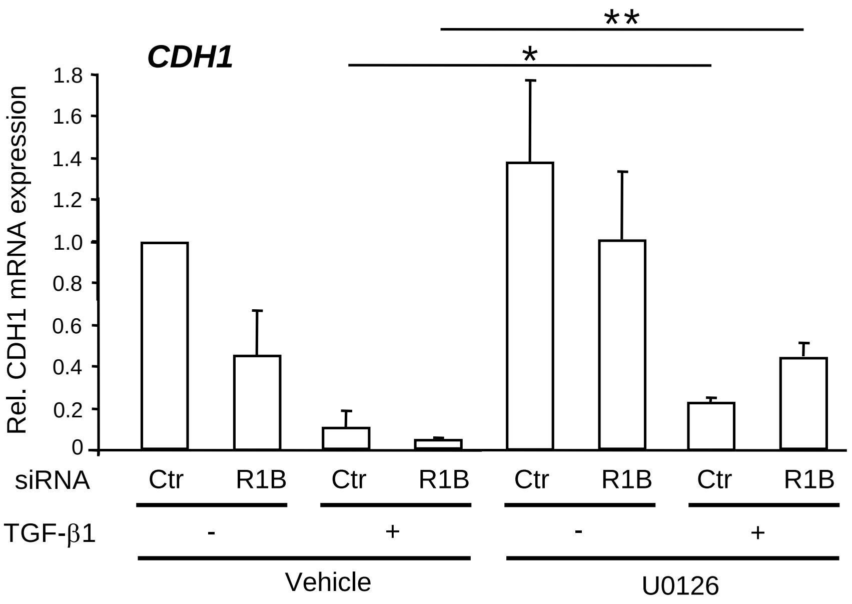

Figure S5. MEK-ERK signaling is involved in TGF-β1 and RAC1B regulation of *CDH1*. Panc1 cells were transfected twice with 50 nM of an siRNA specific to RAC1B (R1B) or a control (Ctr) siRNA, serum-starved overnight and treated with vehicle (0.1% dimethylsulfoxide), or UO126 (10 μM) in the absence or presence of TGF-β1 (5 ng/ml) for a period of 48 h. Cells were then subjected to qPCR analysis of *CDH1*. Data represent the TBP-normalized mean ± SD of three experiments. The asterisks indicate significance.

Supplementary Figure S6

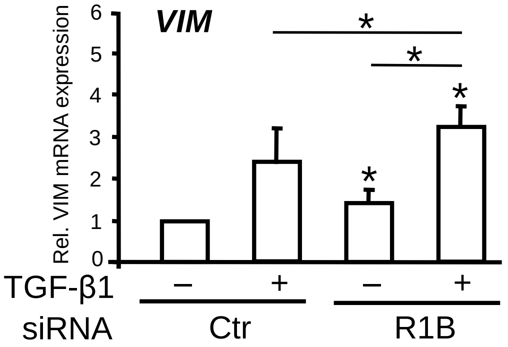

Figure S6. Effect of RAC1B knockdown on *VIM* expression in Panc1 cells. Panc1 cells were transiently transfected twice with 50 nM each of control (Ctr) siRNA or RAC1B siRNA (R1B) and treated or not with TGF-β1 for 24 h. Cells were then processed for RNA isolation and qPCR analysis of *VIM*. Data represent the TBP-normalized mean ± SD of three experiments. The asterisks indicate significance.

Supplementary Figure S7

Full uncropped blots from Figure 1A

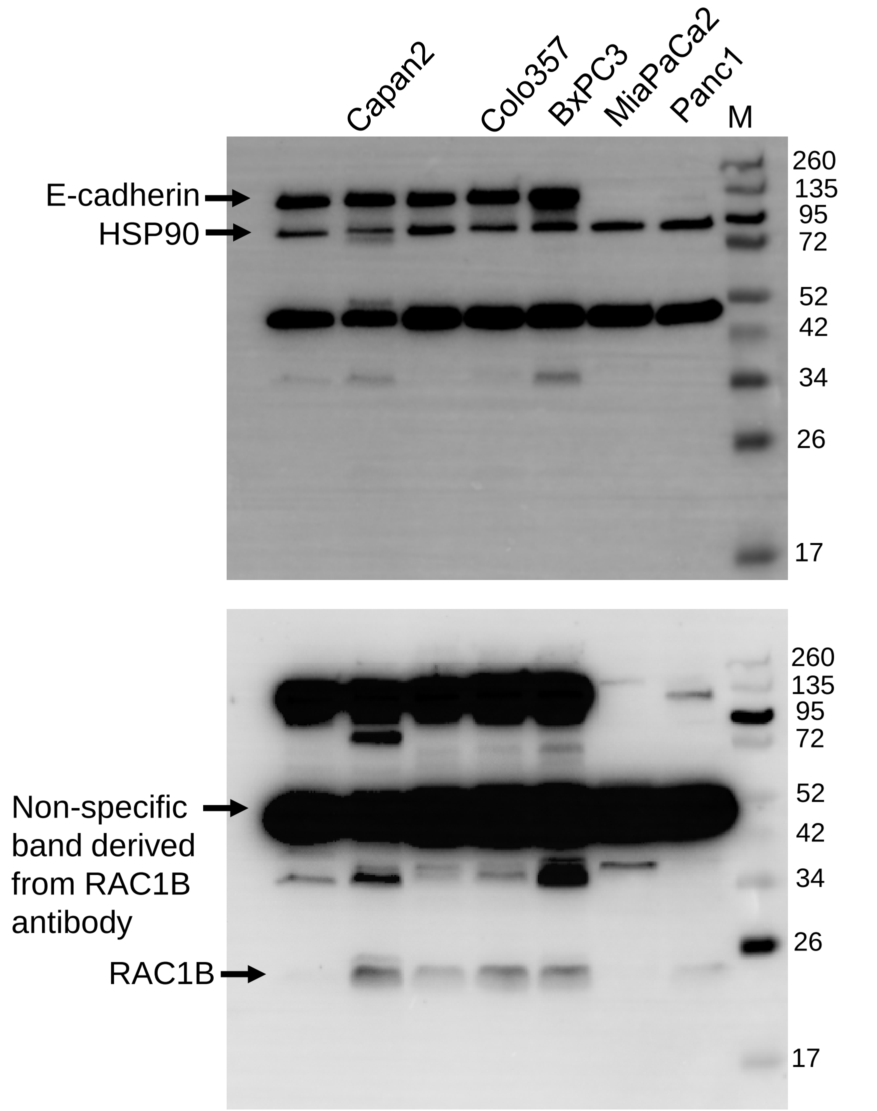

Full uncropped blots from Figure 4A

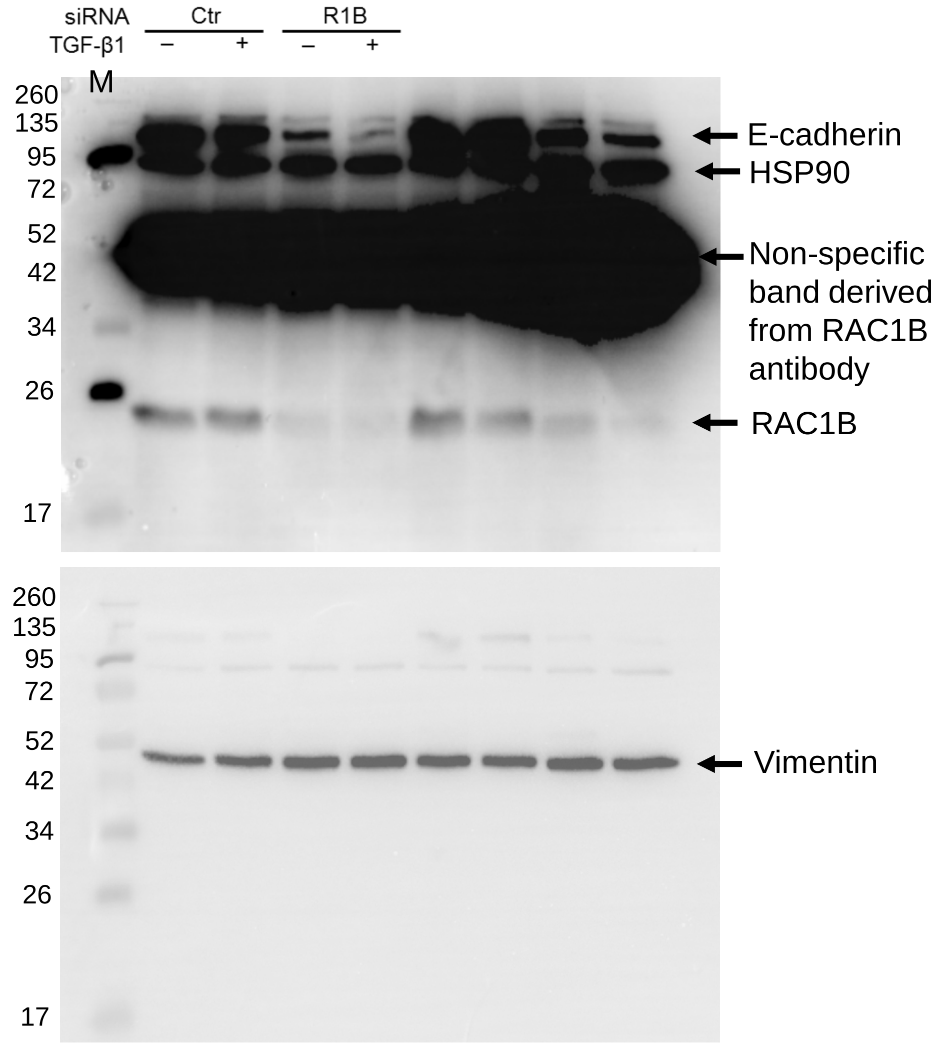

M = molecular weight marker (SM1841, Fermentas/Thermo Fisher Scientific)
